# Comparison of Sleeve Volume Between Banded and Non-banded Sleeve Gastrectomy: Midterm Effect on Weight and Food Tolerance—a Retrospective Study

**DOI:** 10.1007/s11695-022-06404-2

**Published:** 2022-12-12

**Authors:** Mohamed Hany, Bart Torensma, Ahmed Zidan, Ann Samy Shafiq Agayby, Mohamed Ibrahim, Mohamed El Shafie, Iman El Sayed

**Affiliations:** 1 Department of Surgery, Medical Research Institute, Alexandria University, 165 Horreya Avenue, Hadara, Alexandria, 21561 Egypt; 2 Consultant of Bariatric Surgery at Madina Women’s Hospital (IFSO Center of Excellence), Alexandria, Egypt; 3 Clinical Epidemiologist, Leiden University Medical Center (LUMC), Leiden, The Netherlands; 4 Department of Radiology, Faculty of Medicine, Alexandria University, Alexandria, Egypt; 5 Biomedical Informatics and Medical Statistics Department, Medical Research Institute, Alexandria University, Alexandria, Egypt

**Keywords:** Laparoscopic sleeve gastrectomy, Banded sleeve gastrectomy, Sleeve pouch volume, Weight regain, Food tolerance

## Abstract

**Background:**

Sleeve dilatation after laparoscopic sleeve gastrectomy (LSG) causes weight regain (WR). Banded sleeve gastrectomy (BSG) was proposed to prevent dilatation and reduce WR.

**Methods:**

A retrospective cohort study on patients who underwent BSG and LSG and completed 4 years of follow-up from 2016 to 2021 was included. Body mass index (BMI), percentage of excess weight loss (%EWL), percentage of total weight loss (%TWL), and FT scores were calculated at 1, 2, 3, and 4 years. The sleeve volume was estimated at 6 months, 1 year, and 4 years. Multi-variate analysis was conducted to assess correlations between covariates. WR was calculated as weight gain > 10%, > 10 kg above the nadir, or BMI increase of ≥ 5 kg/m 2 above the nadir.

**Results:**

This study included LSG 1279 patients and BSG 132 patients. Mean %EWL at 1 year was 83.87 ± 17.25% in LSG vs. 85.71 ± 7.92% in BSG and was 83.47 ± 18.87% in LSG and 85.54 ± 7.48% in BSG at 4 years. Both had significant weight loss over time (*p*. < 0.001) with no significant main effect of surgery (*p*.0.438). Mean sleeve volume at 6 months was 102.32 ± 9.88 ± 10.28 ml in LSG vs. 101.89 ± 10.019 ml in BSG and at 4 years was 580.25 ± 112.25 ml in LSG vs. 157.94 ± 12.54 ml in BSG (*p*. < 0.001).

WR occurred in 136 (10.6%) and 4 (3.1%) (p.0.002) in LSG and BSG patients, 90 (7%) vs. zero (0%) (*p*.0.002) and 31 (2.4%) vs. zero (0%) (*p*.0.07) using the > 10%, > 10 kg increase above the nadir and the ≥ 5 kg/m 2 BMI increases above the nadir formulas, respectively.

**Conclusion:**

BSG had significantly lower sleeve volume, significantly lower WR, and significantly lower FT scores than LSG after 4 years from surgery; however, volume changes were not correlated with weight loss.

**Graphical Abstract:**

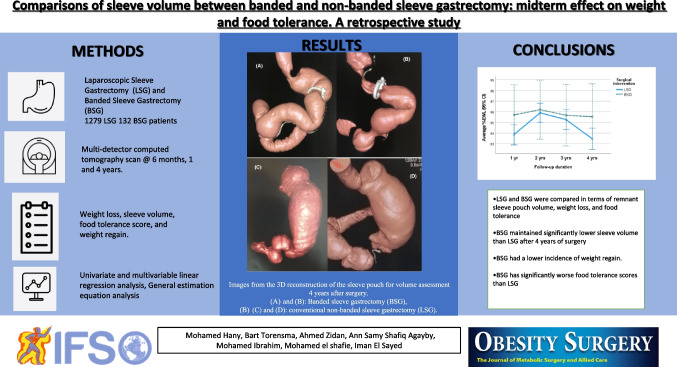

## Introduction

Laparoscopic sleeve gastrectomy (LSG) has remained the most common bariatric procedure worldwide since 2014, according to the IFSO global registry [1]. LSG has a high safety profile with reported lower complications and re-intervention rates when compared to Roux en-Y gastric bypass (RYGB) [2, 3]. Higher rates of insufficient weight loss or weight regain (WR) reaching up to 30% at 5‒7 years after LSG have been reported [4, 5].

Even though the role of sleeve dilatation in WR after LSG is still uncertain, dilatation of the sleeve may be an important cause of WR after LSG as loss of restriction allows more food intake [6, 7]. WR has been reported to have higher revisional surgery rates after LSG than after RYGB, reaching up to 13% [2, 4, 6]. Moreover, up to 4.5% of patients that have undergone LSG need revisional surgery for the dilated sleeve [7].

Some studies proposed banded sleeve gastrectomy (BSG) by applying a band around the upper part of the sleeve pouch to prevent its expansion, hence maintaining better weight loss [8]. Some studies reported significantly higher weight loss in the BSG cohort compared to the non-banded LSG cohort, and some reported weight loss rates after BSG equal to after RYGB [9–11]. Some studies revealed no significant differences between BSG and LSG in weight loss [12]. Furthermore, some reported more eating problems with more regurgitation, vomiting, and dysphagia in the BSG cohort than in the non-banded LSG cohort [9, 11, 13]. Thus, the BSG is supposed to maintain better weight loss due to less sleeve dilatation and less food tolerance (FT).

Radiological assessment of the sleeve volume is a useful tool to report sleeve dilatation accurately [14]. This volumetric assessment can be used to correlate the dilatation and weight loss after LSG and BSG.

The study aimed to compare the outcomes between LSG and BSG in terms of sleeve volume, weight loss, WR, and food tolerance (FT) over an intermediate follow-up period of 4 years. To our knowledge, there are no published studies that compared these, in addition to the objective evaluation of FT.

## Methods

This was a retrospective cohort study whereby the files of all consecutive patients who had primary LSG or BSG at Madina Women’s Hospital, an IFSO-accredited center of excellence (European chapter), the Radiology department at Main University Hospital, and Medical Research Institute, Alexandria University, Alexandria, Egypt, between January 2016 and December 2017 and who completed a follow-up period of 4 years were included in this study.

The details of both procedures were explained to the patients, and they were left to choose. The patients had to pay for the minimizer ring. Bariatric procedures are partly covered by the university, whereby in the other center, the whole procedure was paid for by the patient itself.

Written informed consent was obtained from participants. The study conformed to the principles of the Declaration of Helsinki and was approved by the ethical committee of the Medical Research Institute.

### Study Endpoints

Weight loss, sleeve volume, FT score, and WR.

### Patient Selection

All the patients were preoperatively screened and indicated for bariatric surgery according to the International Federation for the Surgery of Obesity and Metabolic Disorders (IFSO) criteria.

### Data Collection

The analyzed data included demographic characteristics and associated medical problems before surgery, operative time, pre-operative workup, and post-operative follow-up.

### Pre-operative Workup

In all cases, routine laboratory tests and nutritional assessment, upper gastrointestinal endoscopy (UGE), and ultrasound (U/S) abdominal examination were performed. Gastro-esophageal reflux disease (GERD) was evaluated using the Los Angeles classification [15].

### Post-operative Follow-up

Body mass index (BMI), percentage of total weight loss (%TWL), and percentage of excess weight loss (%EWL) were measured at, 1, 2, 3, and 4 years after surgery. %TWL was calculated using the formula: %TWL = (initial body weight − current body weight)/initial weight × 100%. The ideal body weight (IBW) was calculated based on an ideal BMI of 25 kg/m^2^ using the formula: IBW = 25 × (height in m)^2^ [16, 17] %EWL was calculated using the formula: %EWL = (initial body weight − current body weight) / (initial body weight − IBW) × 100%.

### Weight Regain Formulas

WR was defined and tested with three formulas: (1) as a 10% regain of the nadir weight at the last follow-up visit [18, 19]. (2) > 10 kg above the nadir weight [5], and (3) BMI increase of ≥ 5 kg/m^2^ above the nadir [20].

### Food Tolerance

Food tolerance (FT) assessment was performed using the one-page questionnaire to assess the overall patient’s tolerance to food with a score ranging from 1 to 27 at 1, 2, 3, and 4 years after surgery [21].

### Multi-detector Computed Tomography (MDCT)

The sleeve volume was assessed in all patients using an MDCT virtual gastroscopy and 3D reconstruction at 6 months, 1 year, and 4 years after surgery. MDCT Gastrographic has been performed on 64 detectors multi-detector CT scanners (Siemens SOMATOM® Perspective, Siemens Medical Solutions, Malvern, PA). Patients were instructed to fast for at least 4 h before examination and given an intravenous injection of 40 mg butyl-scopolamine and then asked to swallow 2 to 4 packs of effervescent granules (sodium bicarbonate) as tolerated on the table with no water. Performing the MSCT was funded by the university hospital for all the patients (A full description of the technique is in the Appendix) [14].

### LSG Operation

The LSG operation was performed with a standardized technique by two experienced teams of surgeons. The gastric sleeve was created using Echelon Flex Endopath 60-mm linear stapler (Ethicon Endo-Surgery, Cincinnati, OH, USA) starting at 3‒5 cm from the pyloric ring up to the angle of His, over a 40 fr bougie, while paying attention to excise the whole fundus. The staple line was routinely reinforced with seromuscular continuous sutures using barbed 3/0 V-Loc 180 barbed sutures (Covidien, Mansfield, MA, USA).

### BSG Operation

In the BSG, the MiniMizer ring ® (Bariatric Solutions, Stein am Rhein, Switzerland) was applied around the gastric sleeve at 4‒5 cm below the esophagogastric junction, using the blunt built-in needle to pass atraumatically between the lesser omentum vessels, and then the ring was locked at a circumference of 7.5‒8 cm to allow the passage of 5 mm laparoscopic instrument within the ring beside the sleeve with the 40 fr bougie inside the stomach. The ring was fixed by two stitches using Ethibond (Ethicon, Somerville, NJ).

### Hiatal Hernia Repair

Hiatal hernia repair was attempted in cases with pre-operatively diagnosed hiatal hernia during routine UGE. The repair was done using continuous barbed V-Loc™ PBT non-absorbable 2–0 suture (Covidien Medtronic, Mansfield, MA).

### Post-operative Care

Prophylaxis against venous thromboembolism with enoxaparin started 12 h before the operation and continued day 1 after surgery for at least 3 weeks. Patients started a liquid diet on the day of the surgery and were discharged after receiving feeding instructions.

### Statistical Analysis

Quantitative data were described by mean and standard deviation, while categorical data were summarized as frequencies or percentages. Fisher exact (FEp) and Monte Carlo significance (MCp) were performed if more than 20% of the total expected cell counts were < 5. An independent sample *t*-test was performed to compare the mean quantitative variables based on the normal distribution of variables by the Kolmogorov‒Smirnov test and a large sample size > 30 per group.

A mixed design repeated measures ANOVA test was conducted to study if the statistically significant main effect of post-operative time, the main effect of surgery whether LSG or BSG and if an interaction is present in the form of change pattern of the sleeve pouch volume, BMI, %TWL, %EWL, and FT score along different post-operative periods between both two groups.

Predictors were evaluated through univariate and multivariable linear regression analysis. We conducted a multivariate linear regression model using the enter method to assess the independent contribution of parameters such as age, sex, preoperative BMI, diabetes, having ≥ 1 associated medical problem, type of surgery, sleeve volume change from 1 to 4 years, and the interaction between volume change at 1, 2, 3, and, 4 years post-operative follow-up period for %EWL as an outcome variable. We tested the assumptions in terms of linearity by scatter plot; homoscedasticity and normality by residual plot, histogram, normal probability plot, and independence of errors by Durbin‒Watson test. We did not detect problems with multicollinearity for continuous predictors as assessed by correlation matrix, VIF, tolerance, and collinearity diagnostics [20].

General estimation equation analysis was performed to produce unbiased average estimates with 95% confidence intervals (95% *CI*) of %EWL change from baseline among patients who underwent BSG and LSG approaches at 4 years postoperative follow-up period adjusted for the parameters. This method is intended to adjust for correlation due to repeated measurements per participant.

All statistical tests were conducted using IBM SPSS statistics (IBM SPSS Statistics for Windows, Version 28.0. Armonk: IBM Corp.) and R software (Version 4.0.4. package) at 0.05 significance level.

## Results

The data analysis identified 1528 and 152 patients who underwent LSG and BSG, respectively, between January 2016 and December 2017. A total of 1279 (83.7%) LSG patients and 132 (86.8%) BSG patients completed 4 years of follow-up and were included in the study.

### Loss to Follow-up:

A total of 1279 (83.7%) patients with LSG and 132 (86.6%) patients with BSG completed the 4-year follow-up, with a total loss to follow-up rates of 16.3% in the LSG and 13.4% in the BSG cohorts.

### Pre-operative

Table 1 presents the two cohorts’ demographic characteristics and associated medical problems before surgery. There were no significant differences between the two cohorts.

**Table 1:** Comparison of demographic and associated medical problems between patients performing LSG and BSG

	LSG (*n* = 1279)	BSG (*n* = 132)	Sig
Female sex *n* (%)	956 (74.7%)	104 (78.8%)	0.306
Age (years) (mean ± SD)	35.54 ± 10.84)	34.38 ± 9.71	0.239
Preoperative weight (kg) (mean ± SD)	132.99 ± 26.05	133.06 ± 26.36	0.975
Preoperative height(cm) (mean ± SD)	1.67 ± .09	1.67 ± .09	0.919
Preoperative BMI (mean ± SD)	47.62 ± 7.05	47.69 ± 7.13	0.913
Smoking *n* (%)	451 (35.3%)	47 (35.6%)	0.937
Alcohol *n* (%)	18 (1.4%)	2 (1.5%)	0.710
Preoperative associated medical problems:			
*Hypertension n* (%)	387 (30.3%)	40 (30.3%)	0.991
*Diabetes mellitus n* (%)	221 (17.3%)	24 (18.2%)	0.794
*Sleep apnea n* (%)	161 (12.6%)	19 (14.4%)	0.554
*Dyslipidemia n* (%)	386 (30.2%)	40 (30.3%)	0.977
*Osteoarthritis n* (%)	328 (25.6%)	34 (25.8%)	0.978
*Cardiac disease n* (%)	81 (6.3%)	8 (6.1%)	0.908
*Psychological disorders n* (%)	162 (12.7%)	16 (12.1%)	0.858
*Vascular diseases n* (%)	194 (15.2%)	20 (15.2%)	0.966
*DVT, PE n* (%)	20 (1.6%)	2 (1.5%)	1
*Hiatal hernia n* (%)	80 (6.3%)	7 (5.3%)	0.665
*GERD, Grade “A” n* (%)	258 (20.2%)	25 (18.9%)	0.736
Having ≥ one associated medical problems *n*(%)	1080 (84.4%)	104 (78.8%)	0.092

### Outcomes After Surgery

**Fig. 1:**
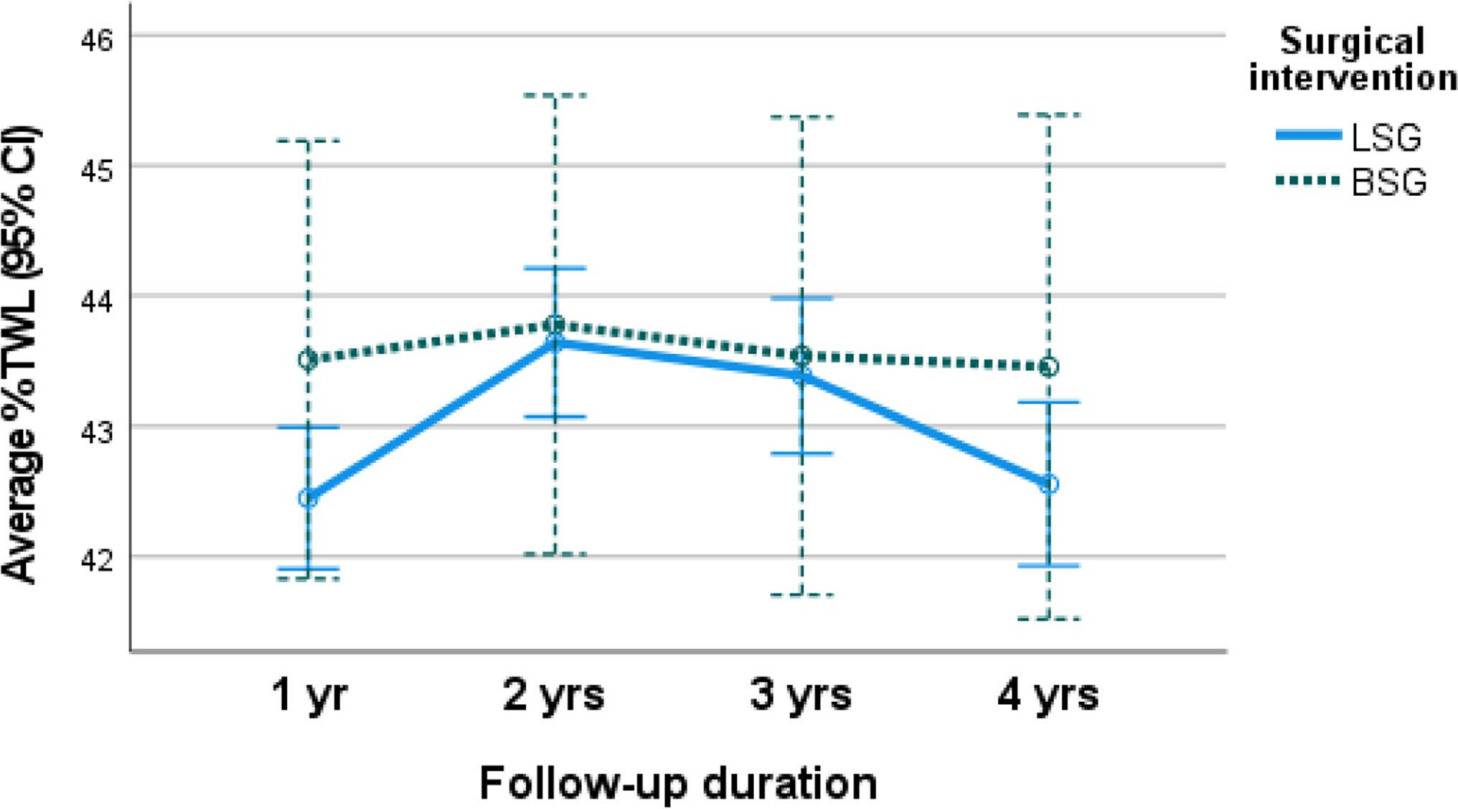
Average percentage of total weight loss (%TWL) in the LSG and BSG cohorts

**Fig. 2:**
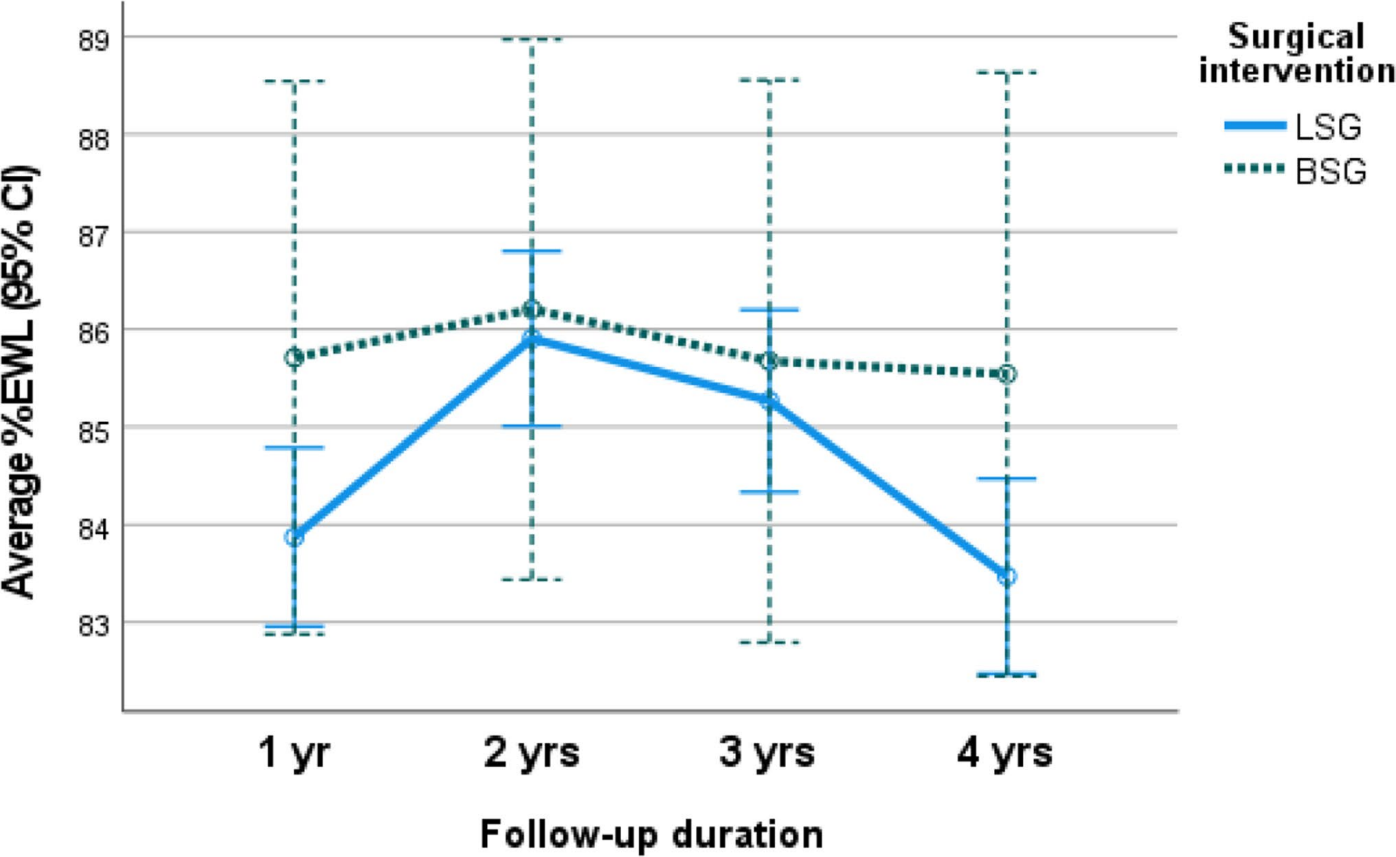
Average percentage of excess weight loss (%EWL) in the LSG and BSG cohorts

The mixed design ANOVA analysis revealed an overall significant increase in the mean %TWL (*p*.0.002) and mean %EWL (*p*. < 0.001) at 1 and 2, 3, and 4 years postoperatively from baseline. The non-significant main effect of the type of intervention was detected with a close increase in mean %TWL and mean %EWL for those who underwent BSG and LSG (*p*.0.550 and 0.438, respectively). Significant interaction between change in mean %TWL and mean %EWL along post-operative follow-up period and type of surgery explained the greater increase in %TWL and %EWL among those who underwent BSG than those who underwent LSG (*p*.0.043 and 0.047, respectively) (Table 2 and Figs. 1 and 2).

**Table 2:** Outcomes after surgery: weight loss, volumetric study, and food tolerance

		*LSG (n* = *1279)*	*BSG (n* = *132)*	*Sig*
BMI (mean ± SD)	*1 yr*	27.05 ± 4.30	26.48 ± 2.04	*p*^a^0.052 *p*^b^ = 0.740 *p*^c^ < 0.116
	*2 yr*	26.38 ± 3.79	26.35 ± 1.86	
	*3 yr*	26.38 ± 4.09	26.45 ± 1.83	
	*4 yr*	26.41 ± 5.20	26.49 ± 1.87	
%TWL (mean ± SD)	*1 yr*	42.45 ± 10.04	43.51 ± 7.62	*p*^a^ < 0.002* *p*^b^ = 0.550 *p*^c^ < 0.043*
	*2 yr*	43.64 ± 10.54	43.78 ± 7.56	
	*3 yr*	43.39 ± 11.00	43.54 ± 7.72	
	*4 yr*	42.56 ± 11.65	43.45 ± 7.68	
%EWL (mean ± SD)	*1 yr*	83.87 ± 17.25	85.71 ± 7.92	*p*^a^ < 0.001* *p*^b^ = 0.438 *p*^c^ = 0.047*
	*2 yr*	85.91 ± 16.87	86.21 ± 7.17	
	*3 yr*	85.27 ± 17.57	85.68 ± 7.30	
	*4 yr*	83.47 ± 18.87	85.54 ± 7.48	
Sleeve volume in ml (mean ± SD)	*6 months*	**(*n* = 1215)** 102.32 ± 9.88	**(*n* = 125)** 101.89 ± 10.09	*p*^a^ < 0.001* p^b^ < 0.001* *p*^c^ < 0.001*
	*1 yr*	**(*n* = 959)** 177.63 ± 10.28	**(*n* = 118)** 110.95 ± 10.09	
	*4 yr*	**(*n* = 911)** 580.25 ± 12.25	**(*n* = 112)** 157.94 ± 12.54	
Food tolerance score (mean ± SD)	*1 yr*	21.46 ± 0.54	21.43 ± 0.62	*p*^a^ < 0.001* *p*^b^ < 0.001* *p*^c^ < 0.001*
	*2 yr*	22.80 ± 0.73	21.54 ± 0.68	
	*3 yr*	23.72 ± 0.86	21.45 ± 0.60	
	*4 yr*	23.81 ± 0.82	21.52 ± 0.56	

Mixed design repeated measures ANOVA test to assess the main effect of time on different parameters,^a^main effect of surgical interventions, ^b^interaction to assess the pattern of change of each quantitative variable along time by surgical intervention. ^c^*Significant results ≤ 0.05

### Sleeve Volume

**Fig. 3:**
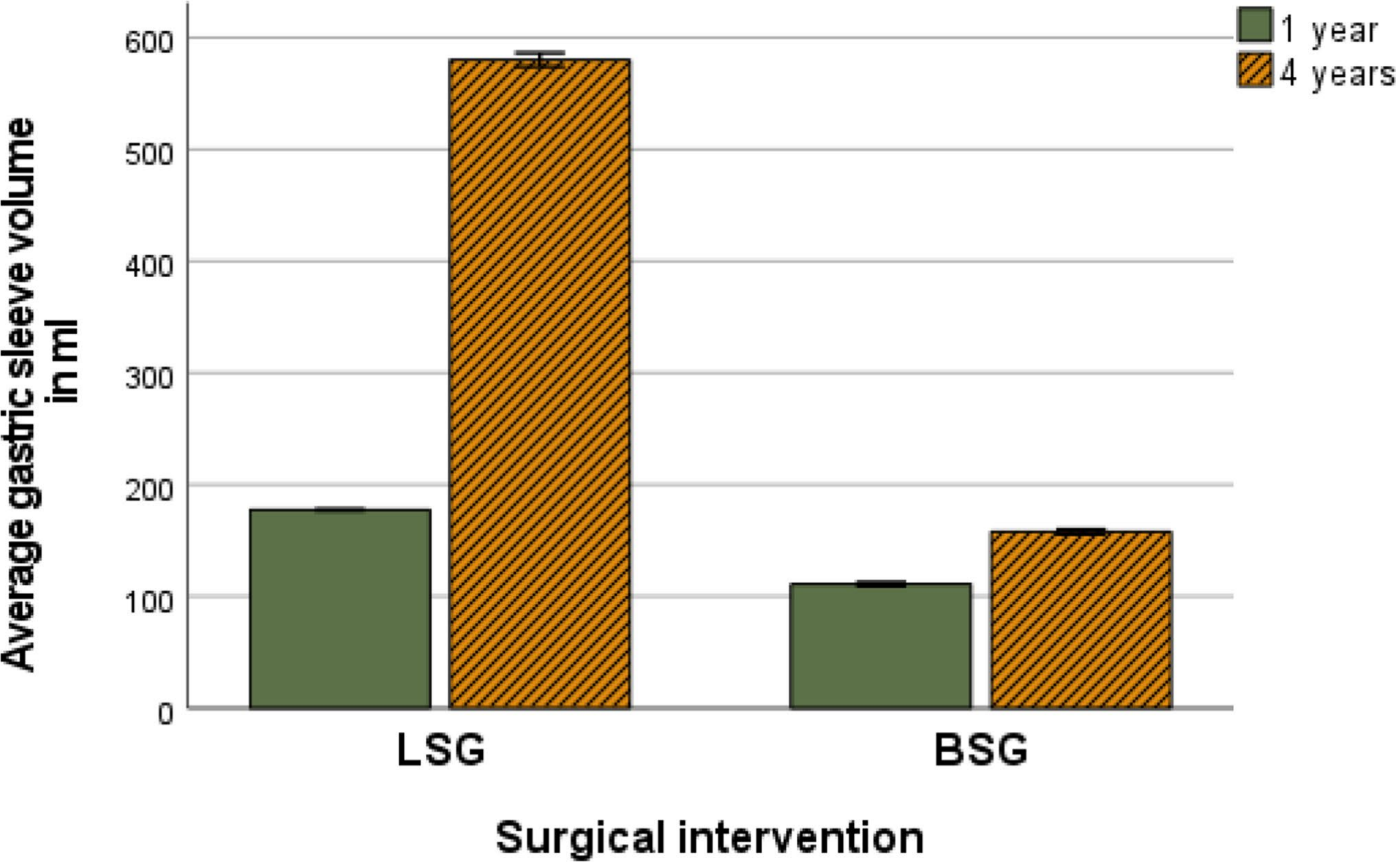
Average sleeve pouch volume in ml in the LSG and BSG cohorts

**Fig. 4:**
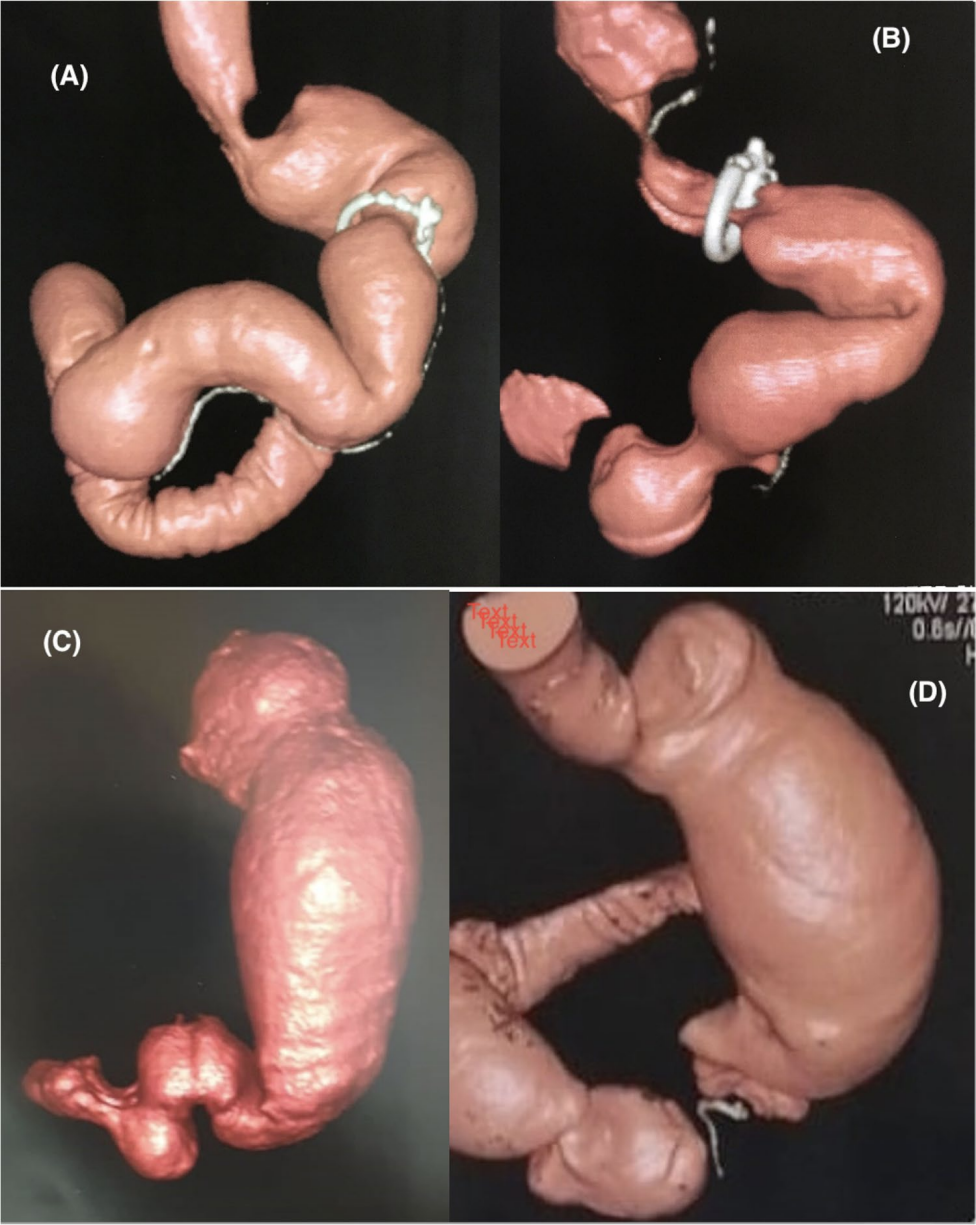
Images from the 3D reconstruction of the sleeve pouch for volume assessment 4 years after surgery. (A) and (B): Banded sleeve gastrectomy (BSG), (C) and (D): conventional non-banded sleeve gastrectomy (LSG). N.B: Hiatal hernia is noted in Figure B.

The mean sleeve volume increased significantly in both cohorts throughout the follow-up (*p*. < 0.001). A significant main effect of the type of surgical intervention was detected with a greater increase in mean volume in the LSG cohort than in the BSG cohort (*p*. < 0.001). A significant interaction denoted a larger increase in mean volume in the LSG cohort than in the BSG cohort (*p*. < 0.001) (Table 2 and Fig. 3). Figure 4 shows images from the 3D reconstruction of the sleeve pouch for volume assessment.

### Food Tolerance

The mean FT score revealed significant improvement in both cohorts throughout follow-up (*p*. < 0.001), with a significant main effect of the surgical intervention (*p*. < 0.001) and a significant interaction (*p*. < 0.001) (Table 2).

### Complications

Three cases of gastric leakage in the LSG group were managed by stent insertion with favorable outcomes. The 30-day-postoperative severe morbidity rates were comparable in the study groups with (*p* = 1.0) [six (0.46%) in LSG and one (0.8%) in BSG]. In the BSG group, 25 patients presented with esophagitis preoperatively, and the postoperative routine endoscopy in 21 of them (84%) showed regression of the reflux. No case of gastric band erosion or slippage was noted in our BSG cohort. Three patients (2.3%) in the BSG group experienced solid dysphagia and reflux symptoms during the follow-up period. Endoscopy revealed constriction at the band site that required a few endoscopic pneumatic balloon dilation sessions with satisfactory results*.*

### GERD

GERD grade “A” was recorded in 258 (20.2%) patients in the LSG cohort, while it was recorded in 25 (18.9%) patients in the BSG cohort (*p*.0.736). (Table 1).

### Operation Time

The operative time was almost equal in both BSG and LSG cohorts; 41.63 ± 7.45 and 41.82 ± 7.55, respectively (*p*.0.953).

### Hiatal Hernia Repair

Hiatal Hernia was diagnosed preoperatively by endoscopy in 80 (6.3%) patients and 7 (5.3%) patients in the LSG and BSG cohorts (*p*.0.665); all patients had a crural repair done during their bariatric procedures (Table 1).

### Weight Regain

In the LSG cohort, weight regain occurred in 136 (10.6%) cases, and the BSG 4 (3.1%) according to the formula > 10% above nadir.

When the formula weight gain of > 10 kg above the nadir was used, the LSG had 90 (7.0%) and BSG zero (0.0%), and when the formula BMI increase of ≥ 5 kg/m^2^ above the nadir, WR occurred in 31 (2.4%) cases in the LSG and zero (0.0%) in the BSG cohort.

WR was significantly higher in the LSG cohort (*p*.0.002) according to the first and second formulas, while according to the third formula, there was no significant difference (*p*.0.07).

### Conversion

No patients from the BSG group needed conversion to another procedure, while 171 patients (13.4%) in the LSG had conversions to RYGB surgery. The indications for conversion were weight regain in 76 (3.9%), GERD in 64 (5.0%), and GERD with WR in 31 (2.2%) patients.

### Multi-variate Analysis

Multi-variate linear regression analysis for %EWL change from baseline has concluded coherent findings with mixed design ANOVA results (Table 3) at 1 and 2 years after surgery.

**Table 3:** Multivariate analysis and general estimation equation for prediction of and EWL% change from baseline adjusted for age, sex, diabetes, having more than one associated medical problem, and body mass index

Covariates	Outcomes		
	Coeff	[95% CI]	Sig
At 1 year: †			
BSG	1.989	[− 1.007 to 4.985]	0.193
Having ≥ 1 associated medical problems	1.921	[− .533 to 4.376]	0.125
Preoperative BMI ≥ 40	− .223	[− 2.738 to 2.292]	0.862
At 2 year: †			
BSG	.450	[− 2.470 to 3.370]	0.762
Having ≥ 1 associated medical problem	2.379	[− .013 to 4.771]	0.051
Preoperative BMI ≥ 40	3.776	[1.325 to 6.227]	0.003*
At 3 years: †			
BSG	.558	[− 2.460 to 3.576]	0.717
Having ≥ 1 associated medical problems	2.830	[.353 to 5.307]	0.025*
Preoperative BMI ≥ 40	5.935	[3.395 to 8.475]	< 0.001*
At 4 years: †, ††			
BSG	2.210	[− 16.520 to 16.851]	0.984
Having ≥ 1 associated medical problems	2.412	[-.300 to 5.123	0.081
Preoperative BMI ≥ 40	8.39	[5.665 to 11.128]	< 0.001*
General estimation equation regression analysis: ¶			
	**Est. Average**	**[95% CI]**	**Sig**
Type of surgery:			
BSG	82.44	[80.16 to 84.72]	0.284
LSG	83.72	[81.51 to 85.92]	
Associated medical problems:			
Having no associated medical problems	82.21	[79.06 to 85.40]	0.059
Having ≥ 1 Associated medical problems	84.46	[82.43 to 86.72]	
Preoperative BMI:			
< 40	80.83	[77.46 to 84.21]	0.007*
≥ 40	85.59	[83.68 to 87.72]	

^*^Significant results ≤ .05

^†^Data indicate changes from baseline to 1, 2, 3, and 4 years. Coefficients of the multiple linear regression analysis go along with 95% confidence intervals (CI). Predictors are age, sex, preoperative BMI, Diabetes, having ≥ one associated medical problem, and type of surgery

^††^prediction of % EWL at 4 years adjusted for age, sex, preoperative BMI, diabetes, having ≥ one associated medical problems, type of surgery, sleeve volume change from 1 to 4 years, and interaction between volume change and surgical intervention type

General estimation equation to predict %EWL change from baseline till 4 years along 1, 2, and 3 years postoperative follow-up period adjusted for age, sex, preoperative BMI, diabetes, having ≥ one associated medical problem, gastric sleeve volume change after 4 years, and type of surgery

Though EWL% change from baseline increased approximately 2%, 0.45%, 0.558%, and 2.2% among BSG than LSG group at 1, 2, 3, and 4 years after surgery, respectively, this difference was not statistically significant.

Patients with preoperative BMI ≥ 40 experienced a significant increase in %EWL compared to those with BMIs < 40 at 2, 3, and 4 years after surgery (3.776, 5.935, and 8.39%, respectively; *p*.003, *p*. < 0.001, *p*. < 0.001, respectively).

General estimation equation regression analysis revealed a comparable estimated increase in %EWL at 4 years postoperative follow-up period from baseline by 82.21% (95% *CI*: 80.16‒84.72%) in BSG compared to the estimated increase of %EWL by 83.72% (95% *CI*: 81.51 to 85.92%) in LSG (*p*.0.284). Patients with preoperative BMI ≥ 40 experienced a significant increase in %EWL by 85.59% (95% *CI*: 83.68‒87.72%) while those with BMI < 40 experienced increases in %EWL by 80.83% (95% *CI*: 77.46‒84.21%) (*p*.0.007) (Table 3).

## Discussion

This retrospective study reported the outcomes between LSG and BSG on weight loss, WR, sleeve volume, and food tolerance (FT) over an intermediate follow-up period of 4 years.

### Weight Loss

In this study, the mean %EWL at one year was 83.87% ± 17.25 in LSG vs. 85.71% ± 7.92 in BSG, and at four years, it was 83.47% ± 18.87 in LSG and 85.54% ± 7.48 in BSG.

Both operations had significant weight loss over time (*p* =  < 0.001), but no significant differences between both surgical interventions (*p* = 0.438).

Two systematic reviews (SR) on %EWL in BSG and LSG reported that at 1 year, the %EWL ranged from 52.4 to 77.4% for BSG and 41.4‒73.5% for LSG, while at 3 years, it ranged from 66.7 to 91% for BSG and 55.9‒73% for LSG, and at 5 years, it ranged from 70.7 to 68.7% for BSG and 53‒57.8% for LSG [8, 22]. Nevertheless, the samples for the BSG in the studies were small (from 13 to 96), and not that many cases were found (6 studies). A recent new SR in 2022 by Ballesteros et al. showed in 7 observational studies and 3 RCTs with a pooled data set of 911 participants in the observational studies and 194 in the RCT that the BSG had a significantly higher %EWL [23]. The evidence suggests that the BSG procedures would favor a higher %EWL; nevertheless, our study was not significantly different on this endpoint.

### Weight Regain

In general, WR after bariatric surgery is one of the main motives for modifying the established bariatric procedures and the evolution of new procedures [8]. Data from systematic reviews showed dilatation of the sleeve pouch as one of the suggested mechanisms of WR. No significant correlation between WR and sleeve pouch dilatation in the literature was found [5, 6].

In this study, WR was calculated using three formulas, (1) > 10%, (2) > 10 kg, and (3) ≥ 5 kg/m^2^ above the nadir weight. In the outcome, the data distribution changed with the different formulas. From the highest incidence with formula 1 (136 vs. 4 cases in LSG and BSG) to lower incidence in the other formulas 2 and 3 (90 vs. 0 and 31 vs. 0 cases). Despite the different incidences, the LSG had significantly more WR after the operation than the BSG. So this suggests that the BSG procedure has equal weight loss after 4 years but prevents better weight regain after the primary procedure and can be seen as an added value for the long term in maintaining a healthy weight.

Interestingly is that different formulas give new insights into the definition and usage of WR. A study by El Ansari et al. in 2021 did a scoping review for weight regain and insufficient weight loss after bariatric surgery. The study concluded that after checking multiple definitions, the inconsistency, multiplicity, and lack of a standardized definition of WR led to poor reporting and understanding of the clinical significance of the WR [6]. Since the international criteria on what the WR formula should be or what the best consensus could be is unclear. A call to action from the bariatric metabolic surgery community could be necessary, whereby new studies on the best validity and predictor should be tested.

### Sleeve Volume

LSG is primarily a restrictive procedure that also involves changes in the gut hormones and microbiomes.

Nevertheless, the loss of restriction by sleeve dilatation has been correlated to WR after restrictive procedures such as LSG and adjustable gastric band (LAGB) when the dilated pouch allows the patient to eat more food [3, 6–8]. A study concluded that sleeve pouch dilatation might lead to WR with a > 4 folds increase of the mean volume of the sleeve pouch from 120 to 524 ml within 5 years in patients who had WR after LSG [3].

Furthermore, in banded RYGB, dilatation of the gastric pouch and the gastro-jejunostomy with the loss of restriction have been attributed to WR [24]. Sleeve pouch dilatation after LSG was correlated to 4.5% of the revisions after LSG [7]. Nevertheless, some studies revealed cases of WR after LSG without sleeve pouch dilatation and others with doubling the sleeve pouch without WR. However, those studies included small size and only followed up for 20‒36 months [25, 26].

This study showed to our knowledge, for the first time, in a large cohort of 911 patients in the LSG, the 3.6-fold increase in the stomach after 4 years (580 ml vs. 157 ml) in LSG and BSG, respectively. However, the multivariate analysis revealed that the changes in the sleeve pouch volume did not correlate to the %EWL after 4 years of follow-up, while having a BMI ≥ 40 was the only predictive factor to have a higher average %EWL. Other studies reported similar results with no correlation between the sleeve pouch volume and weight loss [14, 26, 27]. Nevertheless, the BSG can add value in maintaining stomach volume and preventing WR in the longer follow-up phase since there was significantly more patient in the LSG with increased stomach volume and significantly lower incidence of WR.

### Food Tolerance

Mean FT scores in this study were showing that FT is significantly better in the LSG (*p*. < 0.001) after 4 years (23.81 vs. 21.52).

Generally, LSG has the best-reported FT scores in the bariatric procedures [28]. However, lower FT scores are also expected in the BSG group than in the non-banded LSG group, as studies have reported more regurgitation, vomiting, and dysphagia in the BSG [9, 11, 13].

Laparoscopic adjustable gastric banding (LAGB) has the worst reported FT among bariatric procedures [28]. Moreover, non-banded RYGB has reported significantly better FT total scores than banded RYGB, especially in the first 6 months after surgery [29].

The lower FT score in the BSG confirms the restrictive feeling over time from the band. In contrast, in the LSG, the increasing size of the stomach can give a more comfortable feeling, affecting the patient’s behavior and eating habits. Therefore, on the forehand, good preparation is necessary with good nutritional and psychological consulting for the period after BSG since this can affect the behavior and feelings of the patients. So, it is a good balancing act for which benefits outweigh which. Results clearly show the advantages against WR and stomach volume. However, the long-term slight disadvantages of the FT should not be forgotten.

### GERD

In this study, grade “A” GERD was recorded in 258 (20.2%) patients in the LSG cohort, while it was recorded in 25 (18.9%) patients in the BSG cohort.

It is still debatable if the LSG is a good option for patients with GERD. While some studies reported de novo GERD after LSG, others reported progressive improvement of GERD symptoms over time with weight loss after LSG [30].

During the preoperative consults, the patients from this study chose to proceed with LSG despite being diagnosed with GERD after having a detailed explanation of the advantages and disadvantages of available bariatric procedures. Many patients and surgeons continue to proceed for LSG even with low-grade GERD for its lower morbidity compared to Roux-en-Y gastric bypass (RYGB) [31].

Hiatal Hernia was diagnosed preoperatively by endoscopy in 80 (6.3%) patients and 7 (5.3%) patients in the LSG and BSG cohorts, respectively. Many authors have recommended exploration of the hiatus with crural repair for wide/stretched hiatus [30, 32]. Many studies have reported that patients with diagnosed hiatal hernia who had LSG plus crural repair had higher rates of remission or improvement in the GERD symptoms also lower incidence of de novo GERD compared to patients who had LSG alone [33–36].

### Banded Sleeve Gastrectomy Procedure

This study used the MiniMizer ring ® for its easy deployment and adjustment to lengths from 6 to 8 cm. Different materials have been used to band the sleeve in BSG, including Gortex mesh, silicon bands, Alloderm grafts, and silastic ring [8, 23]. Silastic rings such as the MiniMizer ring ® and GaBP ring™ are the most reported bands with BSG [11].

## Limitations

This study had some limitations as a retrospective study that is inherently inferior to prospective randomized controlled studies with possible undetected confounders or bias that was not corrected or measured. Also, this study did not go in-depth on the incidence of reflux, complications, and associated medical problems post-operatively since this is already discussed in a previous study [37]. Furthermore, an additional limit is the very small size of the BSG group compared to the LSG group (the ratio between BSG and LSG group is 1:10). Therefore, possible complications or findings that are less common could not be found in the BSG group. Larger and funded studies could increase the power of the BSG group; nevertheless, this study is a promising step forward in new techniques around the therapy of patients with obesity.

## Conclusion

BSG prevents sleeve pouch dilatation and maintains significantly lower sleeve volume than LSG. However, the dilatation could not be correlated to the rate of %EWL. BSG was associated with a significantly lower incidence of WR. Moreover, BSG maintained significantly worse FT scores than LSG.

## Data Availability

Data availability is possible on request by the corresponding author.

## Appendix. Multi-detector computed tomography (MDCT)

Image acquisition was performed in the spine position and limited to the stomach, which is adequately inflated with gas on the topogram. Scans were acquired using the least radiation dose with the following parameters: 80 kV, 125 mA, 32 × 0.6 mm collimation, with 1 mm slice thickness reconstruction using SAFIRE iterative reconstruction. Data were transferred to a dedicated 3D workstation. Three-dimensional volume-rendering images were created by a combination of manual and semi-automatic segmentation tools. Different masks were created to represent the various relevant structures in different colors. The volume of the stomach (in preoperative series) and the sleeve pouch was measured on multiplanar reformations. The volume of the resected stomach was estimated by subtracting the pouch volume at 6 months postoperatively from the preoperative stomach volume, putting in consideration that intraoperative sleeve pouch construction was standardized throughout the study. The whole procedure was performed and interpreted in all patients by one radiologist.
